# Conserved Endonuclease Function of Hantavirus L Polymerase

**DOI:** 10.3390/v8050108

**Published:** 2016-05-02

**Authors:** Sylvia Rothenberger, Giulia Torriani, Maria U. Johansson, Stefan Kunz, Olivier Engler

**Affiliations:** 1 Institute of Microbiology, University Hospital Center and University of Lausanne, Lausanne CH-1011, Switzerland; Sylvia.Rothenberger-Aubert@chuv.ch (S.R.); Giulia.Torriani@chuv.ch (G.T.); 2 SIB Swiss Institute of Bioinformatics, Lausanne CH-1015, Switzerland; maria.johansson@isb-sib.ch; 3 SPIEZ Laboratory, Austrasse, Spiez CH-3700, Switzerland

**Keywords:** Bunyaviridae, hantavirus, emerging diseases, endonuclease

## Abstract

Hantaviruses are important emerging pathogens belonging to the Bunyaviridae family. Like other segmented negative strand RNA viruses, the RNA-dependent RNA polymerase (RdRp) also known as L protein of hantaviruses lacks an intrinsic “capping activity”. Hantaviruses therefore employ a “cap snatching” strategy acquiring short 5′ RNA sequences bearing 5′cap structures by endonucleolytic cleavage from host cell transcripts. The viral endonuclease activity implicated in cap snatching of hantaviruses has been mapped to the N-terminal domain of the L protein. Using a combination of molecular modeling and structure–function analysis we confirm and extend these findings providing evidence for high conservation of the L endonuclease between Old and New World hantaviruses. Recombinant hantavirus L endonuclease showed catalytic activity and a defined cation preference shared by other viral endonucleases. Based on the previously reported remarkably high activity of hantavirus L endonuclease, we established a cell-based assay for the hantavirus endonuclase function. The robustness of the assay and its high-throughput compatible format makes it suitable for small molecule drug screens to identify novel inhibitors of hantavirus endonuclease. Based on the high degree of similarity to RdRp endonucleases, some candidate inhibitors may be broadly active against hantaviruses and other emerging human pathogenic Bunyaviruses.

## 1. Introduction

Hantaviruses belong to the *Bunyaviridea* family, a large group of segmented negative strand RNA viruses that include causative agents of severe human diseases [1,2,3]. Hantaviruses merit significant attention as emerging pathogens with expanding global distribution and incidence on the rise [4,5,6,7]. In Asia, the prototypic Hantaan virus (HTNV) and Seoul virus (SEOV) can cause hemorrhagic fever with renal syndrome (HFRS) with fatality rates of up to 3%. In the Americas, the hantaviruses Sin Nombre (SNV) and Andes (ANDV) are associated with hantavirus cardiopulmonary syndrome with up to 40% mortality [7,8,9,10,11,12]. Puumala virus (PUUV) is endemic in Northern Europe where it causes *nephropathia endemica*, a milder form of HFRS, while Dobrava-Belgrade virus (DOBV) is frequently associated with more severe disease [13]. There is currently no licensed vaccine against hantaviruses and therapeutic options are limited. The development of novel strategies for antiviral therapeutic intervention is therefore an urgent need.

Hantaviruses are enveloped viruses with three negative single-stranded RNA segments, small (S), medium (M) and large (L) [12,14]. The S segment encodes the viral nucleoprotein (N), M the precursor of the envelope glycoprotein (GPC) that gives rise to the mature glycoproteins Gc and Gn, and the L segment codes for the viral RNA-dependent RNA polymerase (RdRp), also known as L protein. The polymerase domains of the hantavirus L protein contain the five conserved motifs seen in other viral RNA polymerases [15]. Sequence analysis has revealed a high degree of conservation of the L polymerase domains among hantaviruses and bunyaviruses at large. In analogy to RdRp of other segmented negative-strand RNA viruses, hantavirus L protein functions as an RNA transcriptase and replicase, but lacks “capping activity”, *i.e.*, the capacity to synthesize the 5′cap sequences found in viral transcripts. Similar to RdRp of *Orthomyxoviridae, Arenaviridae*, and *Orthobunyaviridae* hantavirus L protein acquires 5′cap sequence from cellular mRNA transcripts by a mechanism called “cap snatching”. Cap snatching, originally described for influenza virus [16,17], involves binding of the 5′cap structure of a cellular mRNA by the viral RdRp followed by cleavage of the mRNA a few nucleotides downstream of the 5′cap structure by a viral endonuclease activity. The resulting short oligonucleotide bearing a 5′cap is then used by the RdRp as a primer for the synthesis of viral transcripts. In influenza virus, a cap-binding domain was found in the PB2 subunit of the polymerase [18], and an endonuclease domain mapped to the N-terminus of the PA subunit [19,20]. At the structural level, influenza PA endonuclease shares characteristics of the two metal-dependent PD (D/E)X K nuclease superfamily [21] with preference for Mn^2+^ ions [22]. Evidence for cap snatching in bunyaviruses was initially reported more than 30 years ago [23]. Newer studies defined an influenza PA-like endonuclease domain in the N-terminal region of the orthobunyavirus La Crosse (LACV) L protein with structural similarities to influenza virus PA endonuclease [24]. A similar endonuclease activity has recently been identified in the N-terminal domain of hantavirus L protein [25]. Expression of recombinant ANDV L protein resulted in a remarkably high endonuclease activity, which resulted in degradation of viral and cellular mRNAs, including L mRNA itself [25]. Accordingly, expression of ANDV L protein could be rescued upon mutations in the catalytic site of the endonuclease.

Due to their essential role in virus multiplication, the conserved endonucleases of RdRp of segmented negative strand RNA virus polymerases are of great interest for basic virus research. Their nature as enzymes makes them further attractive drug targets for therapeutic intervention. Here, we confirm and extend previous studies, providing further evidence for high structural and functional conservation of endonucleases of geographically distant hantaviruses and Bunyaviruses at large. Based on their known remarkable robust activity, we developed a functional cell-based assay for hantavirus endonucleases that is suitable for high-throughput small molecule screens.

## 2. Materials and Methods

### 2.1. Modeling

The N-terminal sequences of HTNV L and ANDV L polymerase (accession number X55901 and Q9E005_9VIRU, respectively) were compared to the previously characterized N-terminal endonuclease domains of LACV L protein (accession number A5HC98_BUNLC, residues 1–183) and PA influenza virus (PAN) (Influenza A virus A/VietNam/1203/2004 (H5N1), accession number Q5EP34_9INFA, residues one to 209) [24,26]. The active sites of HTNV and ANDV were modeled using the recently determined structure of LACV (PDB entry 2XI5). The suitability of LACV as a template was established through pair wise comparison of profile hidden Markov models (HMM-HMM alignment) using HHpred [27]. The target-template alignments produced by HHpred were manually inspected and modified when necessary. Model structures were calculated using MODELLER [28]. To search for related endonuclease structures in other life forms, we defined structural motifs corresponding to the conserved spatial arrangement of residues of the active site of LACV endonuclease, specifically H34/D79/D92/K94, H34/D52/D79/D92, and H34/P78/D79/D92 [24], with residue numbering according to PDB entry 2XI5. Using DeepView/Swiss-PdbViewer, as described elsewhere [29], we searched for geometric similarities to these motifs in the subset of all protein chains in the RCSB Protein Data Bank (as of 21 October 2014) that originate from non Cα-only X-ray structures with a resolution of at least 3.0 Å and sequence lengths of 40–10,000, for which the maximum pairwise sequence identity between any two chains in the subset is at most 99%. This subset contains ~45,000 chains, from viral, bacterial, fungal, plant, and animal proteins. Our searches did not yield any close geometric matches between the structural motifs and any existing structure in mammalian cells.

### 2.2. Construction of Plasmids

The coding region for HTNV L protein was amplified by PCR using the primers 5′-GGGACTAGTGGCACCATGGATAAATATAGAGAAATTCAC-3′ and 5′-GGGGGATCCATAGAAAGAGGAAATAGAATCCTGC-3′, KAPA HiFi™ DNA polymerase (KAPA BIOSYSTEMS, Wilmington, MA, USA) and pWRG/HTNV-L [30], kindly provided by R. Flick, Ames, Iowa, USA, was used as a template. PCR fragments were subcloned into pCR™-Blunt II-TOPO^®^ vector using Zero Blunt^®^ TOPO^®^ PCR Cloning Kit (Invitrogen™, Carlsbad, CA, USA) according to the manufacturer’s instructions. The fragment comprising the coding region was subcloned into the corresponding sites of the vector pTM, kindly provided by P.Y. Lozach, Heidelberg, Germany. L protein mutants were generated via a classical two-step mutagenesis approach using pWRG/HTNV L as template, the primers containing the desired mutations (all primer sequences are available upon request) and KAPA HiFi™ DNA polymerase (KAPA BIOSYSTEMS). For immunoblotting, hemagglutinin (HA) tag or enhanced green fluorescent protein (EGFP) were fused to the C-termini of the genes. A linker (amino acids sequence G S, nucleotide sequence *Bam*HI, GGATCC) was placed between the coding sequence of the polymerase and the epitope tag. Two sets of L protein constructs were generated, comprising either the full length HTNV L polymerase (pTM-L) or HTNV L endonuclease domain (amino acids 1–223) (pTM-E), generated using the primers 5′-GGGACTAGTGGCACCATGGATAAATATAGAGAAATTCAC-3′ and 5′-GGGGGATCCCTGGCTTTTGTGTGTACTTAT-3′. All plasmids were verified by sequencing. The plasmid pTM-Nluc was generated by PCR using the appropriate primers, KAPA HiFi™ DNA polymerase (KAPA BIOSYSTEMS) and Nanoluc^®^ Luciferase 1.1 (Promega, Madison, WI, USA) plasmid as template. The constructs were verified by sequencing.

To construct the wild-type and mutant L protein fused to nanoluciferase (NLuc), the coding region comprising amino acid 2 to 171 of Nanoluc^®^ Luciferase 1.1 (Promega) gene reporter, flanked by the restriction sites *Bam*HI and *Pac*I was generated by PCR using the appropriate primers, KAPA HiFi™ DNA polymerase (KAPA BIOSYSTEMS) and Nanoluc^®^ Luciferase 1.1 (Promega) plasmid as template. The *Bam*HI-*Pac*I fragment of pTM L-HA and pTM E-HA was subsequently replaced by the *Bam*HI-*Pac*I fragment comprising Nanoluc^®^ Luciferase 1.1 PUUV L protein was isolated from Vero cells inoculated with PUUV. PUUV lot number 612 from National Collection of Pathogenic Viruses (NCPV), Catalogue number 0504101v was cultivated in the biosafety level (BSL) 3 containment facility at Spiez Laboratory, Spiez, Switzerland. After eight days the supernatant was cleared by low-speed centrifugation, virus RNA was extracted using a EZ1 Virus Mini Kit v2.0 (QIAGEN, Hilden, Germany) and reverse transcribed by using random hexamers and the cDNA amplified using virus-specific primers using PrimeScript™ reverse transcription (RT)-PCR Kit (Takara/Clontech, Mountain View, CA, USA). The fragment encoding amino acid 1 to 223 of PUUV L protein was subcloned into pTM as described above using the primers 5′-GGGACTAGTGGCACCATGGAGAAATACAGAGAGATC-3′ and 5′-GGGGGATCCTCGTACTTTTGGGCCTGTGAC-3′. The mutation D97A was generated via a classical two-step mutagenesis approach. The constructs were verified by sequencing.

### 2.3. Cells

BSR T7/5 cells stably expressing T7 polymerase [31] were grown in Dulbecco Modified Eagle Medium (DMEM) GlutaMAX™ (Gibco/Thermo Fisher Scientific, Waltham, MA, USA) supplemented with 10% fetal calf serum (Amimed/BioConcept, Allschwil, Switzerland), 1% Tryptose phosphate Broth 1× (Gibco), 1% MEM non-essential amino acids solution (Gibco) and 1% penicillin streptomycin (10,000 U/mL) (Gibco). Cells were grown in an atmosphere of 5% CO_2_ and 37 °C. Every passage, 1 mg of Geneticin (Promega) per mL medium was added to the culture.

### 2.4. Reporter Assays

Cells were seeded at a density of 2 × 10^5^ cells per well in 24-wells dishes. Transfections were performed using jetPRIME^®^ reagent (Polyplus-transfection Inc., Illkirch, France) or Lipofectamine^®^ 3000 (Life Technologies, Carlsbad, CA, USA). Twenty-four to 48 hours after transfection, cells were lyzed in 1× Cell Culture Lysis Reagent (Promega). The activity of the Nanoluc^®^ Luciferase (Promega) gene reporter was measured using the Nano Glow^®^ Luciferase Assay System (Promega) according to manufacturer’s instructions and using a Lumat LB 9507 (Berthold Technologies, Pforzheim, Germany) luminometer.

### 2.5. Western Blotting

Cell lysates were resuspended in one volume sodium dodecyl sulfate (SDS)-sample buffer and heated at 95 °C for 5 min before being processed by SDS-polyacrylamide gel electrophoresis (SDS-PAGE). Proteins were transferred to nitrocellulose membranes and then detected using specific primary and secondary antibodies. Rat monoclonal antibody 3F10 against HA was from Roche (Basel, Switzerland), mouse monoclonal antibody JL-8 against EGFP from Living Color/Clontech (Mountain View, CA, USA) and mouse monoclonal antibody B5-1-2 against α-tubulin from Sigma (St. Louis, MO, USA). Polyclonal rabbit anti-mouse or anti-rat antibodies conjugated to horseradish peroxidase (HRP) were from Dako (Glostrup, Denmark). Proteins bands were visualized using a chemiluminescence detection kit (WesternBright™ Sirius chemioluminescent HRP substrate (Advansta, Menlo Park, CA, USA) according to the manufacturer’s instructions.

### 2.6. Protein Expression and Purification

The coding region of the N-terminal 220 residues of HTNV L protein (Accession number X55901) was optimized for expression in *E. coli* and synthetized (DNA2.0). Proteins were expressed in *E. coli* strain BL21 (DE3) C41 (Lucigen Coorporation, Middleton, WI, USA) in lysogeny broth (LB) media at 18 °C overnight after induction with 0.2 mM of isopropyl β-D-1-thiogalactopyranoside (IPTG). Cells were lyzed in Tractor buffer (Clontech, Mountain View, CA, USA) supplemented with lysozyme, DNAse, and EDTA-free protease inhibitor cocktail (Roche, Basel, Switzerland).

Proteins from the soluble fraction were purified using a TALON^®^ Metal Affinity resin (Clontech, Mountain View, CA, USA) as recommended by the manufacturer.

### 2.7. In Vitro Endonuclease Assay

For nuclease experiments, 5 to 10 μM of the purified protein were incubated with 25 ng/μL single stranded M13 mp18 DNA (Bayou Biolabs, Metairie, LA, USA) in 20 mM Tris-HCl pH 8.0, 150 mM NaCl, 2.5 mM β-mercaptoethanol and at 37 °C for 60 min. Divalent cations were added to a 2 mM concentration. As a control, the reaction was performed in EDTA 20 mM.

### 2.8. Cell Viability

Cytotoxicity of candidate compounds was assessed using CellTiter-Glo^®^ Luminescent Cell Viability Assay (Promega), which is used to determine the number of viable cells in a culture based on quantification of ATP. Briefly, 2 × 10^5^ cells were plated per well of a 24-well tissue culture plate and transfected with the indicated constructs as described in 2.4. After 24 h, CellTiter-Glo^®^ reagent was added and the assay performed according to the manufacturer’s instructions.

### 2.9. Statistical Analysis

Nanoluciferase assay data were analyzed using one-way analysis of variance (ANOVA) as indicated in the figures and figure legends.

## 3. Results

### 3.1. Conservation of the N-Terminal Endonuclease of Hantavirus L Polymerase

**Figure 1:**
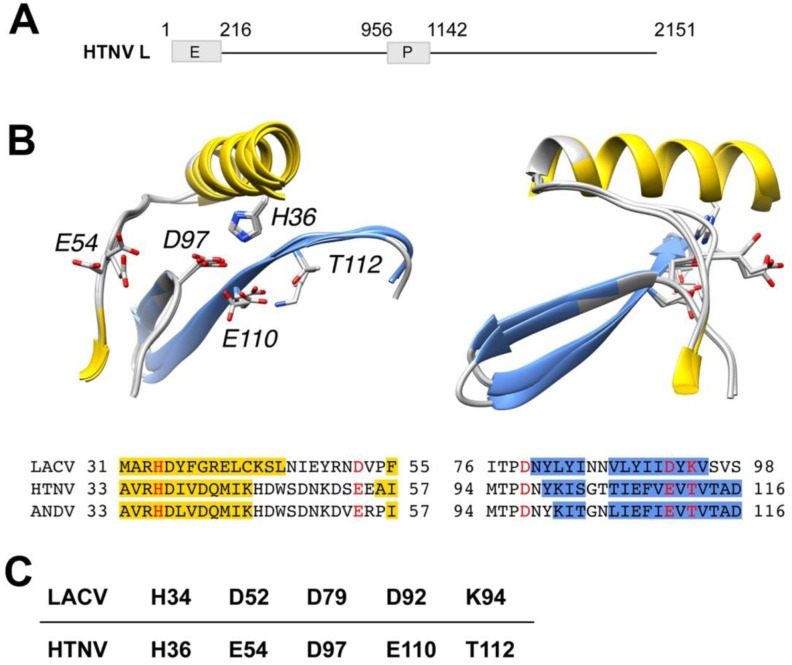
Model of the putative active sites of prototypic Hantaan virus (HTNV) and Andes (ANDV) superposed on the active site of orthobunyavirus La Crosse (LACV). (**A**) Schematic representation of HTNV L protein. The L segment of the prototypic HTNV strain 76/118 has 6533 nucleotides and encodes the L protein of 2151 amino acids (aa). HTNV L contains a polymerase (P) domain (aa 956–1142) and a putative endonuclease (E) domain (aa 1–216). (**B**) Models of the putative active sites of HTNV and ANDV superposed on the active site of LACV. Ribbon diagrams of LACV (PDB entry 2XI5), HTNV and ANDV after structural superposition of key active site residues. Secondary structure classifications was calculated using definition of secondary structure proteins (DSSP) [33] for LACV or predicted using the method/program PSI-PRED [34]. Helix residues have been colored yellow and β-strand residues have been colored blue in the ribbon diagrams as well as in the alignment, according to DSSP or PSI-PRED results, respectively. Annotations in ribbon diagrams follow the HTNV residue numbering in the alignment. Key residues are colored red in the alignment and shown with side chains in the ribbon diagrams. The ribbon diagrams to the right have been rotated 90 degrees around the vertical coordinate axis. (**C**) Amino acid residues of the active site of HTNV L endonuclease derived from the model in (**A**).

Previous studies demonstrated the existence of an endonuclease function in the N-terminal domain of the New World hantavirus ANDV and other hantaviruses [25]. Here we sought to compare the endonuclease function of Old World and New World hantaviruses that differ in geographic distribution, genetics, and disease potential. In a first step, we compared the N-terminal sequences of ANDV L protein to the prototypic Old World hantavirus HTNV and the previously characterized endonuclease domains of LACV L protein (residues 1–183) and influenza virus PD (residues 1–209, H5N1 A/VietNam/1203/2004) [24,26]. As expected, based on the high mutation rate of the viruses, our alignment revealed only low sequence identity of the HTNV L protein N-terminal region with LACV (13.9% at the amino acid level) and with influenza virus PA (9.5%). Sequence homology between the endonuclease domains from LACV and influenza virus was likewise low (8.8%). Secondary structure predictions resulted in a good match between residues 1–183 of LACV L protein and the N-terminal region of HTNV L protein encoded by amino acids 1–220 (data not shown). Next, we modeled the active site of the putative N-terminal endonuclease domains of HTNV and ANDV (residues 1–163) (Figure 1A) using the high-resolution structure of LACV [24] as a starting point, as detailed in Materials and Methods. According to the resulting model shown in Figure 1B, the LACV L endonuclease domain contains a cation-binding fold similar to the one found in influenza virus PA and other members of the PD (*D*/*E*)X K nuclease superfamily. Based on the model, we propose that residues H36, E54, D97, E110 and T112 of HTNV L protein correspond to previously identified key residues of the active site of the LACV endonuclease, namely H34, D52, D79, D92 and K94 (Figure 1B,C). Our structure-based alignment differed only slightly from previous alignments with E54 likely representing a residue within the active site, rather than the originally proposed E75 [24,32]. In sum, our modeling suggested a high degree of conservation of the endonuclease domain between HTNV, ANDV, and LACV.

**Figure 2:**
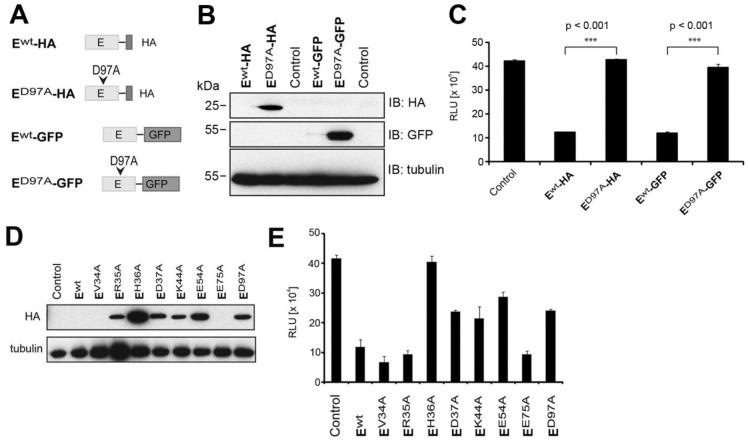
Mutational analysis of HTNV endonuclease domain. (**A**) Schematic representation of the constructs. (**B**) BSR T7/5 cells were transfected with wild-type (wt) or D97A HTNV L protein N-terminal constructs along with an nanoluciferase (NLuc) reporter control plasmid. Cells were lyzed 48 h post-transfection. Proteins were separated by sodium dodecyl sulfate-polyacrylamide gel electrophoresis (SDS-PAGE) and transferred on membranes for immunoblotting. (**C**) NLuc activity was measured using the Nano Glow^®^ Luciferase Assay System (Promega). Data represent mean ± SD (*n* = 3). One representative experiment out of three is shown. Data were analyzed using one-way ANOVA with p-values indicated. (**D**) BSR T7/5 cells were co-transfected with HA-tagged WT L protein N-terminal constructs and the indicated mutants together with an NLuc reporter plasmid. Cells were lyzed 48 h post-transfection and HA-tagged endonuclease constructs detected in Western blot. (**E**) Nanoluciferase activity was measured using the Nano Glow^®^ Luciferase Assay System (Promega). Data represent mean ± SD (*n* = 3). One representative experiment out of three is shown. Data were analyzed using one-way ANOVA with *p*-values *** *p* < 0.001, The significant difference with mutant E75A (*p* < 0.05) was not consistently observed between independent experiments.

To validate our model of the putative active site of the hantavirus endonuclease, we undertook structure function analysis. Expression of recombinant hantavirus endonuclease results in degradation of its proper mRNA, reducing expression to nearly undetectable levels [25]. Mutations of the putative active site restored expression of the recombinant protein [25], allowing the identification of key residues implicated in catalytic activity. Based on the HTNV L protein model (Figure 1B), we generated a recombinant HTNV L protein fragment comprised of the N-terminal 223 amino acids corresponding to the putative endonuclease domain, containing a C-terminal HA-tag or EGFP HTNV Ewt-HA and HTNV Ewt-GFP (Figure 2A). Constructs allowed expression under the control of a T7 promoter allowing efficient expression in the cytosol, where hantavirus replication takes place. In a first step, we mutated residue D97 of HTNV L protein that corresponds to the previously identified residue D79 residing in the PD sequence of LACV L protein [24]. To monitor endonuclease activity of our constructs, we co-transfected a NLuc reporter plasmid, allowing us to measure degradation of reporter transcript via luciferase assay, as previously described [25]. Mutant and wild-type HTNV E-HA and HTNV E-GFP were transiently transfected into BSR T7/5 cells that stably express T7 polymerase. After 48 h of expression, wild-type HTNV Ewt-HA and HTNV Ewt-GFP were barely detectable by Western blot, whereas strong signals were observed for the mutants HTNV ED97A-HA, HTNV ED97A-GFP (Figure 2B). Co-expression of the NLuc reporter construct with HTNV Ewt-HA and HTNV Ewt-GFP, but not the mutants HTNV ED97A-HA and HTNV ED97A-GFP markedly reduced the luminescence signal (Figure 2C).

Next, we subjected HTNV E-HA to site directed mutagenesis performing alanine replacement of additional residues predicted to be located within or close to the putative active site. Specifically, we mutated residues H36, V34, R35, D37, E54, and the previously identified residues K44 [25] and E75 [24,32]. Examination by Western blot revealed that mutations V34A and E75A hardly increased protein expression, suggesting intact endonuclease activity (Figure 2D). In contrast, mutations R35A, H36A, D37A, K44A, E54A, and D97A resulted in at least partial rescue of protein expression, indicating reduced endonuclease activity (Figure 2D). Co-expression of mutants and wild-type HTNV E-HA with the NLuc expression plasmid resulted in reporter activities that correlated with the expression levels of the HTNV E-HA variants (Figure 2D,E).

**Figure 3:**
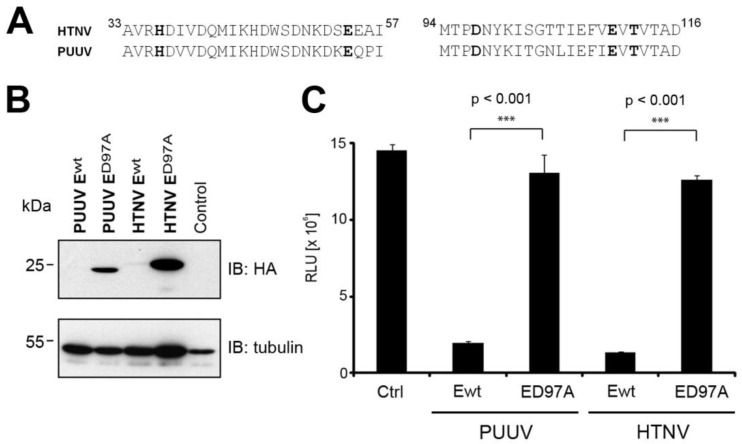
Puumala virus (PUUV) L protein possesses a functional endonuclease domain. (**A**) Sequence alignment of the N-terminal domains of HTNV and PUUV L protein. (**B**) BSR T7/5 cells were transfected with wt or D97A PUUV and HTNV L protein N-terminal constructs along with an NLuc reporter control plasmid. Cells were lyzed 48 hours post-transfection. Proteins were separated by SDS-PAGE and transferred on membranes for immunoblotting. (**C**) NLuc activity was measured using the Nano Glow^®^ Luciferase Assay System (Promega). Data are expressed as mean ± SD (*n* = 3) and were analyzed by one-way ANOVA with p-values indicated.

The remarkably high endonuclease activity of L protein had so far only been demonstrated for hantaviruses associated with severe human diseases. We next compared the endonuclease activity of the pathogenic HTNV with PUUV, which is associated with only mild human disease. At the amino acid sequence level, the putative endonucleases of HTNV and PUUV are similar (Figure 3A), allowing expression cloning of the putative PUUV endonuclease. A side-by-side comparison of the wild-type and D97 mutants of HTNV E-HA and PUUV E-HA revealed similar low expression levels of the wild-type forms with significant rescue upon mutation D97A (Figure 3B). When compared to HTNV ED97A-HA, PUUV ED97A-HA was consistently expressed at lower levels and migrated slightly different. The reasons for this are currently unclear. To quantitatively assess the relative catalytic activity of the endonucleases of PUUV and HTNV, the constructs were co-expressed with our NLuc reporter and lucifease activity detected as described above. The direct comparison showed similar degradation of the NLuc transcript by HTNV Ewt-HA, PUUV Ewt-HA with rescue by the mutation D97A (Figure 3C) indicating that the remarkably high endonuclease activity is conserved between the two hantavirus species.

### 3.2. In Vitro Activity and Cation-Dependence of HTNV L Endonuclease

**Figure 4:**
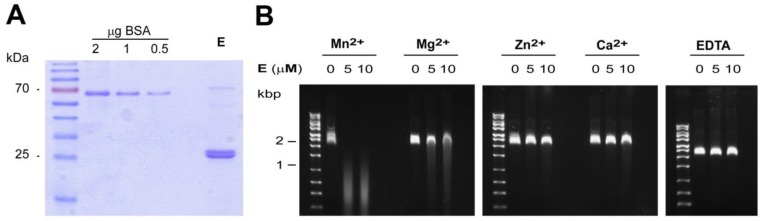
*In vitro* analysis of HTNV endonuclease. (**A**) The N-terminal 220 residues of HTNV L protein were expressed in *E. coli* and purified using TALON^®^ metal affinity resin as described in the methods section. A fraction of the purified material was separated by SDS-PAGE and stained with Coomassie blue. (**B**) Divalent cation-dependent nuclease activity. Single-stranded M13mp18 DNA (25 ng/μL) was incubated at 37 °C during 60 min in presence of 0, 5 or 10 μM of purified HTNV N-terminal domain and 2 mM of the indicated divalent cations, or 10 mM ethylenediaminetetraacetic acid (EDTA).

Biochemical studies on the viral endonucleases of influenza virus and LACV *in vitro* revealed enzymatic activity against single-stranded-RNA (ssRNA) and ssDNA with strong dependence on Mn^2+^ [19,22,24,26]. In order to demonstrate catalytic activity of HTNV endonuclease *in vitro* under defined conditions, we expressed the 220 N-terminal residues of HTNV L protein containing the putative endonuclease domain in a bacterial system as detailed in Section 2.6. As a control, we engineered a “catalytic dead” version. Upon induction of bacterial expression both the wild-type protein and the catalytically inactive mutant were initially produced. However, during subsequent purification, the catalytically inactive variant was consistently lost, resulting in markedly reduced yields. A possible reason may be an overall reduced stability of the protein due to the presence of four mutations H36A, E75A, D97A and E110A, but this remains speculative at this point. The wild-type protein was purified via immobilized metal affinity chromatography (IMAC), resulting in a >90% pure protein as detected by Coomassie brilliant blue (Figure 4A). Using ssDNA, previously identified as a suitable substrate for both influenza and LACV endonucleases [19,24,26], we were able to detect the catalytic activity of our recombinant HTNV endonuclease *in vitro* (Figure 4B). Addressing divalent cation-dependence, we found high activity of HTNV endonuclease against ssDNA in presence of Mn^2+^, partial activity in presence of Mg^2+^, but none upon addition of Zn^2+^, or Ca^2+^ (Figure 4B). Double-stranded DNA (dsDNA) was found to be a poor substrate, excluding non-specific nuclease contamination (data not shown). Together, our *in vitro* studies revealed for the first time enzymatic activity of HTNV endonuclease with a strong preference for Mn^2+^ shared with the endonucleases of LACV and influenza. The low residual activity of HTNV endonuclease in presence of Mg^2+^ resembles influenza PA endonuclease and differs from LACV [22,24]. The reasons for these differences are unknown but may be related to the specific nature of the metal-binding residues in hantaviruses *vs.* orthobunyaviruses.

### 3.3. Development of a Cell-Based Assay for Hantavirus Endonuclease

**Figure 5:**
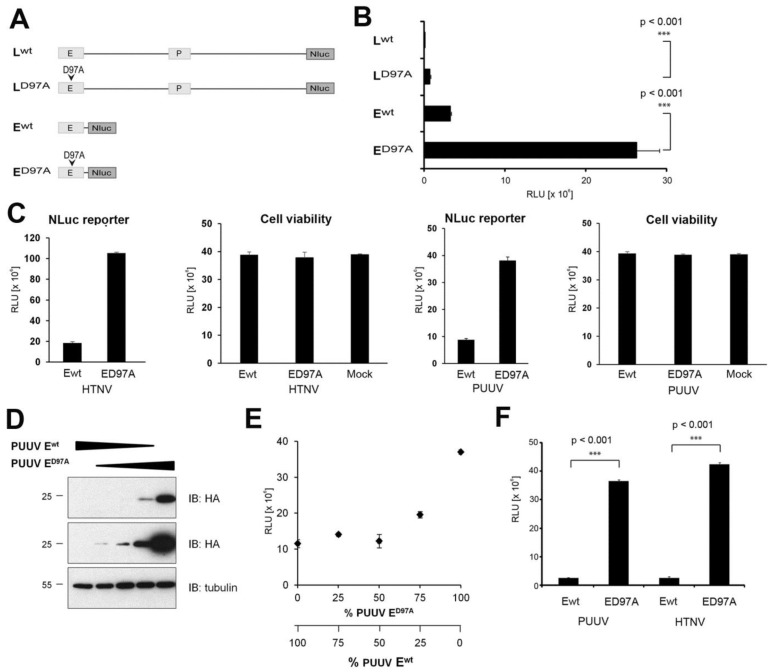
Development of a cell-based assay for endonuclease activity. (**A**) Schematic representation of the constructs. Full-length L protein wt and D97A mutant, as well as the endonuclease domain (E) wt and D97A mutant were fused to NLuc at the C-terminus. (**B**) The constructs were expressed in BSR T7/5 cells. Cells were lyzed 48 h post-transfection and NLuc activity measured using the Nano Glow^®^ Luciferase Assay System (Promega). Data represent mean ± SD (*n* = 3) and were analyzed by one-way ANOVA. One representative experiment out of three is shown. (**C**) Cell viability assay. The indicated NLuc fusion constructs were transfected into BSR T7/5 cells. After 24 h, NLuc reporter activity was measured as in (**B**). Cell viability was assessed by Cell TiterGlo^®^ assay. Data represent mean ± SD (*n* = 3). (**D**) Different relative amounts of PUUV Ewt-HA and PUUV ED97A-HA were co-transfected into BSR T7/5 cells. At 24 h post transfection, protein expression was detected in Western blot as in Figure 2B. (**E**) Different relative amounts of PUUV Ewt-NLuc and PUUV ED97A-NLuc were co-transfected into BSR T7/5 cells and NLuc reporter activity detected after 24 h as in (**B**). Data represent mean ± SD (*n* = 3). (**F**) PUUV Ewt and HTNV Ewt and the corresponding D97A mutants were fused to NLuc at the C-terminus. All constructs were expressed as in (**B**). Cells were lyzed 24 h post-transfection and NLuc activity measured. Data represent mean ± SD (*n* = 3) and were analyzed by one-way ANOVA with *p*-values indicated. One representative experiment out of three is shown.

The remarkably robust endonuclease activity of hantavirus L protein resulting in degradation of transcripts in *cis* and in *trans* opened the possibility to establish a cell-based functional assay for the endonuclease activity based on an NLuc reporter. Previous studies revealed that the efficiency of degradation of mRNAs by hantavirus endonucleases seems proportional to the length of the transcript [25]. We hypothesized that longer transcripts may therefore enhance the sensitivity of our assay. To test this possibility, we fused wild-type and endonuclease dead (D97A) full-length HTNV L protein and HTNV Ewt at the C-terminus to NLuc, resulting in the constructs HTNV Lwt-NLuc, LD97A-NLuc, Ewt-NLuc, and ED97A-NLuc (Figure 5A). Constructs were expressed via a T7-driven cytoplasmic expression plasmid in BSR T7/5 cells. Due to the lack of a specific antibody to NLuc, we were unable to detect our NLuc fusion proteins in Western blot. We therefore relied on the more sensitive NLuc reporter to detect the presence of our fusion proteins. As expected, we observed marked reduction of the NLuc reporter signal in presence of active endonucleases (Figure 5B). The presence of the mutation D97A increased the NLuc signal in the context of the full-length L protein construct HTNV Lwt-NLuc *vs.* LD97A-NLuc consistently by >40-fold, whereas *circa* 8-fold enhancement was observed with HTNV Ewt-NLuc compared to ED97A-NLuc (Figure 5B). The *circa* 5-fold difference in degradation between full-length L protein and the isolated endonuclease domain correlated well with the relative length of their transcripts (Figure 5A). Taken together, our results show that both endonuclease and NLuc activities are maintained in our fusion constructs. The mutation D97A located in the active site of the endonuclease domain has more impact in the context of the full-length L protein polymerase than in the context of the N-terminal domain, likely due to the different length of the mRNA, in line with previous studies [25]. Although the dynamic range of the assay based on the full-length polymerase seemed higher, the absolute signal intensity was considerably lower, resulting in more variability. We therefore opted for the shorter E-NLuc construct for further development of the assay.

A possible concern was unspecific toxicity of our hantavirus E-NLuc construct due to its capacity to degrade cellular transcripts. To address this issue, we expressed wild-type and D97A mutant E-NLuc constructs of HTNV and PUUV under assay conditions for 24 h and assessed cell viability by Cell TiterGlo^®^ assay that detects cellular ATP levels. As shown in Figure 5C, none of the constructs caused significant reduction in cell viability under these conditions. The reduced expression of reporter plasmids co-transfected with wild-type endonuclease (Figure 5C) suggests that the endonuclease has no absolute specificity for viral RNA and may degrade cellular transcripts as well. Of note, in our assay format, expression of wild-type endonuclease was only of short duration (24 h). At this point, the impact on cell viability was only mild (Figure 5C). However, prolonged expression of high levels of recombinant wild-type endonuclease may be detrimental for cells and the duration of the assay has to be kept at a minimum to prevent unspecific off-target effects. A crucial aspect of a functional cell-based assay is its dose–response characteristic. To get a first estimate of the dynamic range of our assay, we transfected cells with different ratios of wild-type and D97A mutant PUUV E-HA and E-NLuc constructs and determined protein expression and reporter activity, respectively. As expected, increased proportion of the D97A mutant construct progressively rescued protein expression of the PUUV E-HA constructs (Figure 5D). Detection of reporter activity in cells transfected with different ratios of wild-type and D97A mutant PUUV E-NLuc resulted in a dose–response characterized by a negligible increase of NLuc activity up to a wild-type to D97A mutant ratio of *circa* 1. With wild-type to D97A mutant ratios of >1, the reporter activity increased inversely proportional to the amount of residual wild-type, as expected (Figure 5E). This particular dose–response characteristic was observed independently of the absolute amount of constructs expressed, suggesting an effect of the ratio, rather than expression level.

In a last step, we assessed the robustness of our assay. To this end, we determined its Z′ value (Z′ = 1 − (3σ_c+_ + 3σ_c−_)/(μ_c+_ − μ_c−_)), which depends on the sum of the standard deviations of positive and negative controls (σ_c+_ and σ_c−_ respectively) as well as the difference between the mean activity of these controls (μ_c+_ and μ_c−_) (35). For the determination of the Z′ value of our assay, wild-type and D97A mutant E-NLuc constructs of HTNV and PUUV were used as positive and negative controls, respectively (Figure 5E). Data from three independent experiments performed in triplicates yielded Z′ values of >0.9 (Table 1), indicating sufficient robustness for implementation in high-throughput formats [35].

Calculated values of the Z′-factor from three independent experiments performed in triplicates. The Z′-factor was calculated as described in Zhang *et al.* [35].

## 4. Discussion

In the present study, we confirmed and extended previous studies providing evidence for a high conservation of the endonuclease activity found in the N-terminal domain of the L polymerase of hantaviruses. We expressed active recombinant hantavirus endonuclease and were able to show catalytic activity *in vitro* and specific preference for divalent cations. Based on its remarkably robust activity, we developed a cell-based functional assay for hantavirus endonuclease suitable for high throughput formats.

Based on the existing high-resolution structure of the LACV endonuclease domain [24], we modeled the active sites of the putative endonuclease domain of HTNV and ANDV L polymerases. The overall fold of hantavirus endonuclease resembled the structure of the LACV homologue, which was expected based on the predicted similar secondary structures and our modeling approach. According to the model, residues H36, E54, D97, E110 and T112 of HTNV L protein correspond to the key residues of the LACV endonucleases active site, H34, D52, D79, D92 and K94. Performing structure–function analysis, using site-directed mutagenesis, we validated our model of the active site demonstrating a crucial role of residues H36, E54 or D97 for the activity of HTNV L endonuclease, in line with previous studies performed on the endonuclease of ANDV [25]. Residues H36, E54, D97, E110 and T112 are fully conserved in all known hantavirus isolates, suggesting that this activity is highly conserved.

The past years have seen the advent of efficient rescue systems for several bunyaviruses [36,37,38,39,40,41]. However, for hantaviruses, progress has been limited to minireplicon systems [30,42] and to the best of our knowledge, no hantavirus rescue system has so far been reported. The robust endonuclease activity of recombinant hantavirus L protein results in degradation of viral and cellular transcripts in *cis* and *trans* [25]. As pointed out earlier [25], the resulting inability to express recombinant hantavirus L protein in sufficient quantities in mammalian cells may represent an obstacle for the development of hantavirus reverse genetics systems. In our present study, we identified mutants of L protein’s endonuclease with partially reduced activity. Such variants may retain sufficient endonuclease activity to fulfill essential functions required for transcription, e.g., cap-snatching, without causing excessive degradation of viral and cellular transcripts. We are currently testing this approach in the context of a minireplicon system.

The robust endonuclease activity of hantavirus L protein resulting in degradation of its own transcript was observed with the N-terminal domain alone and in the context of the full-length L protein. However, it is unclear if L protein reaches sufficiently high expression levels in infected cells to cause a similar effect. Interestingly, recent insights into the structure of the bunyavirus polymerase revealed that the complementary 3′ and 5′ ends of the viral RNA do not form a “panhandle” structure in the pre-initiation complex, as previously anticipated, but are bound to separate sites within L protein distant from the N-terminal endonuclease domain [43]. In the proposed model of viral RNA replication, the 3′ and 5′ termini remain bound to l protein at all times, whereas nascent viral RNA is protected by association with the N protein [43]. If this model of L protein is applicable to hantaviruses as well, L protein’s endonuclease would be unable to degrade viral RNA as long as it remains associated with the polymerase or N. The endonuclease activity of L protein’s N-terminal domain may specifically degrade “naked” viral RNA that may leak out of the replication-transcription complex and may otherwise interfere with efficient replication.

Many potent antiviral drugs currently used for treatment of human infections with negative stranded RNA viruses target viral replication and transcription. The most important drug target within the viral replication-transcription complex is the viral polymerase, which frequently shows a high degree of conservation within a given virus family and catalyzes both transcription and replication. Considering its crucial role in viral transcription and its nature as an enzyme, the endonuclease activity found in RdRp of segmented negative strand RNA viruses appears to be a possible drug target for therapeutic intervention. The data at hand suggest a high degree of conservation of the endonucleases found in RdRp of hantaviruses, bunyaviruses, and orthomyxoviruses. The identification of broadly-active inhibitors of viral endonucleases by small molecule drug screens appears therefore conceivable. Considering the existence of endogenous endonucleases in human cells, whose function is essential for normal physiology, possible cross-reactivity of candidate inhibitors is a concern. To address this potential pitfall, we performed extensive searches based on structural motifs defined by the conserved spatial arrangement of residues of the active site of LACV as detailed in Materials and Methods. Screening of >100,000 structures of proteins from all kingdoms of life, we found a large distance between the conserved viral endonucleases and any mammalian structure, suggesting that the identification of specific inhibitors that block the activity of viral endonuclease, but not human counterparts may be feasible. The main purpose of our current screening assay is to identify specific inhibitors that block the viral endonuclease with minimal cross-reactivity with endogenous human endonucleases. However, the differential inhibition of viral *vs.* human endonuclease would of course represent an essential step of counter-screening following the identification of initial candidate inhibitors that block the viral endonuclease.

Based on the conserved robust endonuclease activity of hantavirus L protein, we developed a reliable cell-based reporter assay suitable for high-throughput screening of collections of synthetic compounds in a rapid and cost-effective manner. The negligible toxicity of our reporter constructs minimizes unwanted off-target effects and therefore the probability of false positive hits. We examined robustness of our assay and found a Z′-value of 0.9, whereas Z′-values > 0.5 are generally considered as “excellent assays” with a low probability of false positive or negative hits [35]. The high throughput format of our assay will allow us to cover large “chemical space”, opening the possibility to identify candidate inhibitors with broad activity targeting conserved structures present in viral endonucleases with possible therapeutic application against a range of known and newly emerging viruses. In addition, candidate small molecule inhibitors identified in our studies may serve as “molecular probes” to dissect the largely unknown mechanisms underlying hantavirus multiplication in human cells and advance our knowledge about this important emerging virus family.

## Figures and Tables

**Table 1:** Evaluation of the quality of the cell-based endonuclease assay.

Experiment	PUUV	HTNV
Number 1	0.94	0.93
Number 2	0.93	0.90
Number 3	0.94	0.90
